# Seasonal fluctuations of small mammal and flea communities in a Ugandan plague focus: evidence to implicate *Arvicanthis niloticus* and *Crocidura spp.* as key hosts in *Yersinia pestis* transmission

**DOI:** 10.1186/s13071-014-0616-1

**Published:** 2015-01-08

**Authors:** Sean M Moore, Andrew Monaghan, Jeff N Borchert, Joseph T Mpanga, Linda A Atiku, Karen A Boegler, John Montenieri, Katherine MacMillan, Kenneth L Gage, Rebecca J Eisen

**Affiliations:** National Center for Atmospheric Research, 3090 Center Green Drive, Boulder, 80301 CO USA; Division of Vector-Borne Diseases, Centers for Disease Control and Prevention, 3156 Rampart Road, Fort Collins, 80522 CO USA; Department of Epidemiology, Johns Hopkins Bloomberg School of Public Health, 615 N Wolfe St, Baltimore, MD 21205 USA; Uganda Virus Research Institute, Entebbe, Uganda

## Abstract

**Background:**

The distribution of human plague risk is strongly associated with rainfall in the tropical plague foci of East Africa, but little is known about how the plague bacterium is maintained during periods between outbreaks or whether environmental drivers trigger these outbreaks. We collected small mammals and fleas over a two year period in the West Nile region of Uganda to examine how the ecological community varies seasonally in a region with areas of both high and low risk of human plague cases.

**Methods:**

Seasonal changes in the small mammal and flea communities were examined along an elevation gradient to determine whether small mammal and flea populations exhibit differences in their response to seasonal fluctuations in precipitation, temperature, and crop harvests in areas within (above 1300 m) and outside (below 1300 m) of a model-defined plague focus.

**Results:**

The abundance of two potential enzootic host species (*Arvicanthis niloticus* and *Crocidura spp.*) increased during the plague season within the plague focus, but did not show the same increase at lower elevations outside this focus. In contrast, the abundance of the domestic rat population (*Rattus rattus*) did not show significant seasonal fluctuations regardless of locality. *Arvicanthis niloticus* abundance was negatively associated with monthly precipitation at a six month lag and positively associated with current monthly temperatures, and *Crocidura spp.* abundance was positively associated with precipitation at a three month lag and negatively associated with current monthly temperatures. The abundance of *A. niloticus* and *Crocidura spp.* were both positively correlated with the harvest of millet and maize.

**Conclusions:**

The association between the abundance of several small mammal species and rainfall is consistent with previous models of the timing of human plague cases in relation to precipitation in the West Nile region. The seasonal increase in the abundance of key potential host species within the plague focus, but not outside of this area, suggests that changes in small mammal abundance may create favorable conditions for epizootic transmission of *Y. pestis* which ultimately may increase risk of human cases in this region.

## Background

The plague bacterium, *Yersinia pestis,* has a global distribution, being found in Africa, Asia and North and South America. However, within this broad geographic range, *Y. pestis* is restricted to foci that are ecologically conducive to the long-term maintenance of transmission cycles [1]. Theories diverge on the exact mechanisms by which *Y. pestis* is maintained and the factors responsible for epizootic spread [2,3]. One theory is that *Y. pestis* is maintained in enzootic cycles by host species populations that display a heterogeneous response to infection. Fleas become infected by the susceptible hosts that develop very high bacteremias required to infect fleas, while the populations of resistant hosts provide the necessary blood meals for fleas to survive, serve to slow the rate of transmission, and possibly give birth to a mixture of resistant and susceptible offspring [2,3]. When *Y. pestis* spills over to highly susceptible host populations (often referred to as epizootic host species), the infection can spread rapidly at epizootic rates (R_0_ > 1) [2]. To date, there is very little empirical evidence from temperate plague foci in North America to support the enzootic host theory (reviewed by [3]). Alternatively, *Y. pestis* could persist within highly susceptible host populations in a metapopulation structure without the need for additional “enzootic” or “reservoir” hosts. Studies from temperate foci in North America and Central Asia have shown support for this metapopulation hypothesis [4-8]. Fundamental to both hypotheses is the notion that contact rates between fleas and hosts dictate the rates of spread of plague bacteria within host communities. Geographic foci and the timing of epizootics are often predicted based on temperature and precipitation patterns, as these factors affect vector and host abundance and contact rates [9-14].

Typically, human infections arise following epizootics when susceptible hosts die of plague infections and their potentially infectious fleas increase their feeding rates on humans [15]. In recent decades, the majority of human plague cases have been reported from tropical plague foci, especially those in east and central Africa and Madagascar where few studies have attempted to identify how plague bacteria are maintained during inter-epizootic periods (but see [16-19]). Such information is fundamental to designing strategies to disrupt the enzootic transmission cycle and ultimately reduce human plague cases.

Our study focuses on the West Nile region of Uganda, which represents an epidemiological focus for plague in that country. Previous studies defined this human plague focus based on environmental predictors [13,14,20,21]. Specifically, areas above 1300 m, which are generally wetter compared with localities below this threshold, pose a greater risk for plague. Furthermore, satellite-derived variables that were significantly associated with elevated risk were consistent with areas where crops are grown seasonally. Within the focus, inter-annual variation in plague case counts was strongly associated with rainfall [22]. Particularly, the number of plague cases in the West Nile region was negatively associated with dry season rainfall (December-February) and positively associated with “intermediate” season rainfall (June-July), prior to the main rainy season from August through November during which most plague cases occur.

Within the West Nile focus and other tropical plague foci in East Africa, little is known about how *Y. pestis* is maintained during inter-epizootic periods and then amplified during epizootics or why the spatial and temporal distributions are strongly associated with rainfall. A recent study showed that although host abundance and diversity were similar inside and outside of a plague focus in West Nile, flea diversity was higher within the plague focus compared with outside and suggested that increased flea diversity aids in long term maintenance of *Y. pestis* [23]. In the same study, flea diversity in the West Nile region was strongly and positively correlated with rainfall amounts. Such a finding is consistent with a dominant enzootic host theory in the East African literature positing that *Y. pestis* is maintained in enzootic cycles by sylvatic hosts (e.g., *Arvicanthis niloticus, Mastomys spp.,* and *Crocidura spp.*) and their fleas with occasional spill over to highly susceptible roof rats, *Rattus rattus*, that live in close association with humans [1,18,19,24,25]. Because of their close association with humans and the high efficiency with which their primary fleas (*Xenopsylla cheopis*) transmit plague bacteria, roof rats (*R. rattus*) and their fleas are believed to serve as bridging species to humans [1,3]. However, the ability of *Y. pestis* to persist within other tropical foci with very low host and vector diversity begs the question of whether separate enzootic hosts are for the maintenance of *Y. pestis*. Indeed, in the highlands of Madagascar plague bacteria are maintained by a single rodent host, *R. rattus*, and two flea species [17,26].

In this study, we collected small mammals and their fleas along an elevation and climatic gradient that included areas within and outside of a previously defined plague focus in the West Nile region, whereby the high elevation regions are inside the focus, and low elevation regions are outside [13]. We examined seasonal changes in the abundances of several potential enzootic plague hosts both inside and outside the plague focus. Our objectives were to identify small mammal and flea species that might play a significant role in *Y. pestis* transmission and to model changes in the seasonal abundance of these key species based on rainfall fluctuations and agricultural practices.

## Methods

### Description of study region

The study was conducted in the Arua and Zombo districts in the plague-endemic West Nile region of northwestern Uganda. The majority of human plague cases in this region are reported from villages located on the Rift Valley escarpment in the western portion of these two districts rather than from the eastern portion below the escarpment [20]. Previous studies have identified 1300 m as a threshold, above which there is an elevated risk of plague in the West Nile region [13,14,20]. Ten small mammal sampling sites were established along a roughly east–west transect that spanned 725 to 1630 m in elevation. The first six sites were below the 1300 m threshold and the last four sites were above 1300 m. Temperature and precipitation vary significantly along the study transect, with higher elevations experiencing cooler, wetter conditions, lush vegetation, and fertile soils, while the lower elevations below the escarpment have sparser vegetation and sandier soils [13]. Additional ecological characteristics of the study region have been described previously in detail [13,14,20,21,25,27].

**Figure 1:**
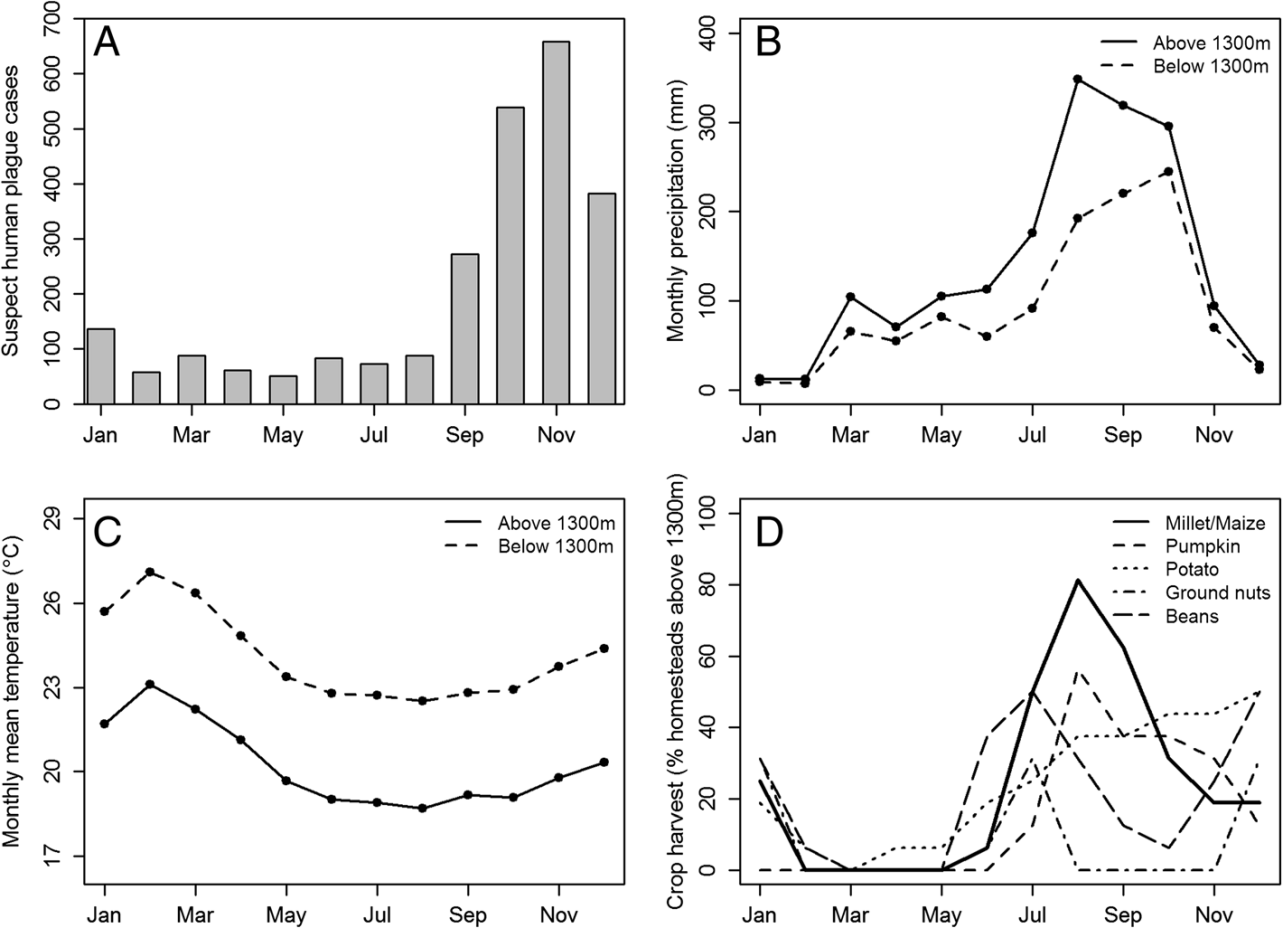
**Monthly plague cases, precipitation, temperature, and crop harvests in West Nile region of Uganda. (A)** Number of monthly suspected human plague cases in Arua and Zombo districts from 1999 to 2011. Averaged monthly **(B)** precipitation (mm) and **(C)** mean temperatures (°C) at field sites above and below 1300 m. Climate variables are long-term averages for 1999–2008 derived from Weather Research and Forecasting model simulations described in Monaghan et al. [35]. **(D)** Percentage of homesteads above 1300 m that report harvesting millet/maize, potatoes, pumpkins, beans, and ground nuts in each month.

A total of over 2,400 suspect human plague cases have been reported in the Arua and Zombo districts since 1999 [22]. The timing of human plague cases is highly seasonal in these districts, with the majority of cases typically arising between September and December each year (Figure 1A). Precipitation in the region is weakly bimodal with an initial rainy season from March through May followed by the primary rainy season from July/August through November (Figure 1B). Temperature also varies seasonally, although these variations are relatively minor compared to the seasonal fluctuations in precipitation. The highest monthly mean temperature occurs in February during the dry season and the lowest monthly mean temperature occurs in August (Figure 1C). Therefore, the annual peak in plague cases coincides with the wettest, coolest time of year, with rainfall during the dry season and in June and July between the two rainy seasons being the most important climatic driver of the year-to-year variability of plague cases in the region [22].

### Description of small mammal and flea collection

**Figure 2:**
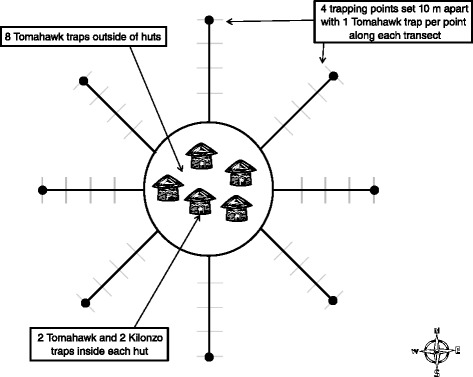
**Schematic of sampling design for a single homestead within a village.**

Collections were conducted during eight separate trapping sessions between February, 2010 and March, 2012. Trapping sessions occurred on a roughly seasonal basis with three sessions occurring in February-March (2010, 2011, 2012) at the end of the dry season, one session during the secondary rainy season in May-June (2011), one session at the start of the primary rainy season in August-September (2011), and three sessions in either October (2010) or November-December (2010, 2011) near the peak or latter portion of the primary rainy season. During each trapping session small mammals and fleas were collected from four homesteads at each of the ten sites along the study transect. Each homestead consisted of a cluster of 2–6 traditional huts with earthen floors, mud waddle walls and thatch roofing. Two Tomahawk and two Sherman traps were placed inside each hut within a homestead. In addition, 16 traps were placed within the family compound outside the huts, and 64 traps were set in the peridomestic area surrounding each homestead by placing two traps (one of each type) at 10 m intervals along eight 40 m-transects extending away from the homestead in the four cardinal and four inter-cardinal directions (Figure 2). For each session, trapping was conducted for two consecutive nights and traps were inoperable from early morning until dusk. Each of the eight trapping sessions included 48–192 trap nights per site within the huts (depending on the number of huts per homestead), 128 trap nights per site immediately outside of the homesteads and 512 trap nights per site along the transect lines away from the homesteads. Across the entire study there were a total of 62,136 trap nights (10,936 inside and 51,200 outside). Fleas were preserved in 70% ethanol and subsequently identified to species using published taxonomic keys [28-31]. All captured small mammals were measured, weighed, identified to species based on morphological measurements [32], and combed for fleas. Small mammal species that could not be easily identified morphologically to species were only identified to genus initially (e.g., *Crocidura spp.*, *Mastomys spp*., and *Praomys spp*.). To confirm field identifications and to describe the species composition of small mammals identified as *Mastomys* spp. and *Crocidura* spp., molecular identifications were made by querying Cytochrome *b* gene sequences against the NCBI nucleotide collection database. During small mammal trapping efforts, ear tissue samples from each individual were collected in 70% ethanol and stored at ambient temperature for up to 4 years. A subsample of specimens were selected for molecular identification so that multiple samples were analyzed for each trapping site and for each separate trapping session to detect any spatial or temporal differences in species identification.

Nucleic acids were extracted from 37 Crocidura and 36 Mastomys tissue samples using the DNeasy Blood and Tissue kit following manufacturer’s protocols (Qiagen, Valencia, CA). Amplification of the ~1140 bp cytochrome *b* gene was achieved using the universal mammalian primers L14723 and H15915 [33]. Each 50 μl PCR reaction contained GoTaq Green Master Mix (1x, Promega, Sunnyvale, CA), 5 μl (5–25 ng) template, and a final concentration of 600 nM for each primer. Cycling parameters have been described previously [34]. Amplified products were purified using the QIAquick PCR Purification Kit (Qiagen, Madison, WI) prior to sequencing on a Dyad DNA Engine thermal cycler using BigDye Terminator v3.1 Ready Reaction reagents (Applied Biosystems, Foster City, CA). Each reaction was then processed using the Big Dye Xterminator Purification Kit (Applied Biosystems) and run on a 3130XL genetic analyzer (Applied Biosystems). Forward and reverse sequences for each sample were assembled, and nucleotide positions for which we did not obtain at least double coverage were manually trimmed from either end of the consensus sequence using Lasergene software (version 11.2.1, DNASTAR, Madison, WI). The resulting sequence was used to query the NCBI nucleotide collection database using the Basic Local Alignment Search Tool (BLAST). Sequences were obtained for 31 *Crocidura* spp. and 30 *Mastomys* spp. samples. Samples were excluded if more than two attempts to extract DNA failed, or if double coverage of at least 750 nucleotides within the target sequence could not be obtained. *Crocidura* spp. sequences ranged in length from 1129 to 1144 bp and all (31 of 31) most closely matched *Crocidura olivieri* cytochrome *b* sequences within the GenBank database (EU742597.1, KC684161.1; Query coverage: 99–100%, query identity: 98–99%). Sequences obtained from individuals identified as *Mastomys* spp. were between 758 and 1136 bp in length. The majority of the samples (20 of 30) were most similar to cytochrome *b* sequences of *Mastomys erythroleucus* (JQ735643.1, GQ227889.1, JX292863.1, JX292868.1, FN393048.1; Query coverage: 98–100%, query identity: 95–99%). Four samples were most similar to *Mastomys natalensis* (HE864089.1; Query coverage: 98–99%, query identity: 99%). The remaining samples (6 of 30) were likely misidentified in the field, and were most similar to cytochrome *b* sequences in the database for *Zelotomys hildegardeae* (n = 3, EU349791.1; Query coverage: 100%, query identity: 98%), *Praomys daltoni* (n = 1, JQ735733.1; Query coverage: 100%, query identity: 97%), *Praomys jacksoni* (n = 1, AF518361.1; Query coverage: 100%, query identity 99%), and *Otomys irroratus* (n = 1, JF619553.1; Query coverage: 100%, query identity: 87%).

### Climate and environmental variables

Due to the lack of ground-based meteorological stations within the study region, and to account for variation in climate variables that arise over a diverse landscape, we used the Weather Research and Forecast (WRF) model to simulate a climate dataset centered over the West Nile region of Uganda [14,35]. WRF is a meso-scale numerical weather simulation model suitable for both research and prediction applications with the ability to resolve small-scale phenomena [36]. WRF simulations were conducted for the period from 1999 to 2008 at a 2-km spatial resolution (WRF simulation details in [35]). Ten-year average monthly precipitation totals and monthly mean temperatures were then calculated for use in our analysis and extracted for each homestead using ArcGIS 10.1 (ESRI, Redlands, CA). Examination of the total rainfall and normalized difference vegetation index (NDVI, a proxy of vegetation biomass) for 2010–2012 indicates that rainfall and vegetation were nearly the same as the 2001–2010 mean during the majority of the eight collection periods, and therefore the 1999–2008 climate data are representative of the conditions during the field study. Exceptions occurred during the February-March collection periods in all three years: rainfall in 2010 was 45–60% above normal, and in 2011 and 2012 was 45–60% below normal. However February-March is a relatively dry time of year, so in absolute terms these percentage departures from normal are small. This fact is evident from the NDVI data, which indicates vegetation biomass was less sensitive to the large fluctuations in February-March rainfall: NDVI was only 15–25% above normal in 2010, and 0–15% below normal in 2011 and 2012. We conclude that the 1999–2008 climate data—which span approximately the same period as the 2001–2010 mean—are representative of the conditions during the field study and are therefore appropriate for use in this study.

A survey of which crops were grown and when they were harvested was conducted at 37 of the 40 homesteads. Eight different crops were planted by over 50% of the homesteads surveyed, and of these eight crops six were harvested seasonally rather than year-round (Figure 1D; beans, potato, maize, pumpkin, millet, and ground nuts). Because millet was harvested by fewer than 50% of the homesteads within the area of elevated risk and maize and millet tended to be harvested at the same time, we combined the millet and maize harvests into a single indicator variable. For each of the other four seasonally variable crops we created indicator variables to designate the months when each homestead typically harvests a particular crop. Full details of the homestead survey results are presented in [37]. All protocols were reviewed by the Science and Ethics Committee of the Uganda Virus Research Institute, the Uganda National Council for Science and Technology, and the Institutional Animal Care and Use Committee (Protocol number 09–023) of the Centers for Disease Control and Prevention’s Division of Vector-Borne Diseases to ensure compliance with Animal Welfare Regulations (9 CFR Chapter 1, Subchapter A, Part 2) and/or the Public Health Service Policy on Humane Care and Use of Laboratory Animals Section IV.

### Data analysis

The abundance of each small mammal and flea species at each of the four homesteads within a trap site was calculated by correcting for trapping effort (number of trap nights per homestead) per trapping session. Host species richness was calculated for each homestead as the number of unique small mammal species (or genera) collected per session and host species diversity was calculated using Simpson’s diversity index [38]. Because several small mammals could be identified only to genus rather than species, we calculated host richness and diversity using both the number of species and number of genera. The results of all analyses were similar using either measure, so only the results using host species are presented here. Flea species richness and diversity were calculated using the same methods based on the fleas collected on-host.

To examine whether host and flea communities differed in and around homesteads inside or outside of the region of elevated plague risk we classified trapping sites by whether their elevation was above or below the 1300 m threshold (4 sites were above 1300 m, 6 below). Each trapping session was classified as occurring during the plague season (from August through December) or outside of the plague season (January through July) in order to examine seasonal variations in the host and flea communities. Elevation differences in host and flea communities were tested via repeated measures analysis by comparing values above or below 1300 m with a generalized linear mixed model using the lme4 package in R [39] to account for the unbalanced number of observations above and below 1300 m. Trapping session and the elevation indicator variable were treated as fixed effects and homestead ID was treated as a random effect. Seasonal differences were also tested using a generalized linear mixed model with the plague season indicator variable treated as a fixed effect and homestead ID as the random effect. We also examined differences in the total abundance of small mammals, the abundance of the four most commonly captured small mammals (*Rattus rattus, Arvicanthis niloticus, Crocidura spp.,* and *Mastomys spp.*), the pooled abundance of small mammals other than the four most common, and flea abundance per host corrected for trapping effort. For each of these variables we examined differences in mammals or fleas collected from within huts only, outside of huts only, or both within and outside of the huts together. The ratio between small mammal abundances at particular locations along the traplines (10, 20, 30, and 40 meters from homestead) were also tested to determine whether small mammals were more likely to be captured closer or further from the homestead during particular months of the year.

The relationships between seasonal fluctuations in the abundance of the most common small mammal species and environmental conditions were explored using linear regression via generalized linear mixed models with trapping site treated as a random effect. For each species whose abundance varied significantly between trapping sessions during and outside of the plague season we examined the relationship between abundance per trapping session and climatic variables at sites above or below 1300 m. Monthly precipitation and mean temperatures for zero to eight months lag were considered as potential explanatory variables to examine the potential for a delayed biological response to climate as predicted by the trophic cascade model [9,10]. In addition, the harvesting of beans, potato, pumpkin, ground nuts, and a combination of maize and millet 0–8 months prior were considered as potential explanatory variables. The best model for each response variable was selected using Akaike’s Information Criterion (AIC) and an R^2^ statistic was calculated from likelihood ratio statistics and only models with an R^2^ > 0.30 were retained [40]. The relationship between flea abundance per host and environmental conditions was examined using the same methods. All analyses were conducted using R 2.15 (R Core Team 2013).

## Results

### Summary of small mammal and flea data

**Figure 3:**
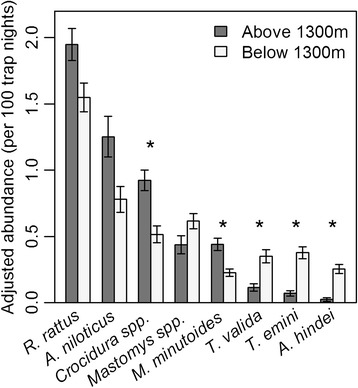
**Abundance (per 100 trap nights) above and below 1300 m of the eight most common small mammals.** Error bars represent ±1 SE. *Indicates statistically significant difference (α = 0.05) between abundance above and below 1300 m using repeated measures analysis with a generalized linear mixed model.

A total of 3,201 small mammals were captured over the eight trapping sessions, with 1,127 (35.2%) collected from within huts, 78 (2.4%) collected from within the family compound outside of the huts, and 1,996(62.4%) collected from the peridomestic setting surrounding the homesteads. Corrected for trapping effort this represents 10.3 small mammals per 100 trap nights within huts, 0.8 per 100 trap nights in the family compound, and 4.9 per 100 trap nights along the traplines. The four most commonly collected hosts (*R. rattus*, *A. niloticus*, *Crocidura spp*., and *Mastomys spp*.) accounted for 2,443 (76%) of all small mammals collected (Figure 3). The majority of small mammals captured inside of the huts were *R. rattus* (993 of 1,127 [88.1%]), and only 83 *R. rattus* were captured outside of the huts. Overall, at least 16 different small mammal species from 13 genera were collected (Table 1). A total of 3,975 fleas comprising at least 12 different species were collected from all small mammals, with over half collected from *A. niloticus* (27%) and *R. rattus* (26%). Over 97% of the fleas collected (3,874 of 3,975) were one or another of the seven most common flea species: *Xenopsylla cheopis, X. brasiliensis, X. nubica, Dynopsyllus lypusus, Ctenophthalmus cabirus, Stivalius torvus,* and *Echinophaga gallinacea* (Tables 1 and 2).

**Table 1:** **Flea infestation of small mammals collected at all ten sit** **es from February 2010 to March 2012**

		**Total no. fleas collected (no. fleas/hosts examined**													
**Host species**	**No. host**	**Xc**	**Xb**	**Xn**	**DI**	**Cc**	**St**	**Eg**	**Cb**	**Cf**	**Dlo**	**La**	**Tp**	**Un**	**Total**
R. rattus	1076	548(0.51)	237(0.22)	7(0.01)	12(0.01)	12(0.01)	4(0.00)	176(0.16)	3(0.00)	25(0.02)	0(0.00)	1(0.00)	1(0.00)	9(0.01)	1035(0.96)
A. niloticus	607	466(0.77)	109(0.18)	7(0.01)	255(0.42)	224(0.37)	2(0.00)	0(0.00)	16(0.03)	1(0.00)	6(0.01)	0(0.00)	0(0.00)	7(0.01)	1093(1.80)
Crocidura spp.	424	329(0.70)	19(0.04)	0(0.00)	25(0.06)	11(0.03)	181(0.43)	0(0.00)	0(0.00)	0(0.00)	1(0.00)	0(0.00)	0(0.00)	6(0.01)	572(1.35)
mastomys spp.	336	323(0.96)	57(0.17)	12(0.04)	26(0.08)	10(0.03)	28(0.08)	0(0.00)	2(0.01)	1(0.00)	0(0.00)	0(0.00)	0(0.00)	5(0.01)	464(1.38)
M. minutodies	193	6(0.03)	0(0.00)	0(0.00)	0(0.00)	0(0.00)	13(0.07)	0(0.00)	0(0.00)	0(0.00)	0(0.00)	0(0.00)	0(0.00)	0(0.00)	19(0.10)
T. valida	158	36(0.23)	1(0.01)	215(1.36)	8(0.05)	0(0.00)	0(0.00)	1(0.01)	0(0.00)	0(0.00)	0(0.00)	0(0.00)	0(0.00)	2(0.01)	263(1.66)
T. emini	157	10(0.06)	0(0.00)	168(1.07)	3(0.02)	0(0.00)	0(0.00)	0(0.00)	0(0.00)	5(0.03)	1(0.01)	0(0.00)	0(0.00)	0(0.00)	187(1.19)
A. hindei	101	200(1.98)	0(0.00)	2(0.02)	2(0.02)	0(0.00)	1(0.01)	0(0.00)	0(0.00)	0(0.00)	0(0.00)	0(0.00)	0(0.00)	0(0.00)	205(2.03)
L. flavopunctatus	61	0(0.00)	8(0.13)	0(0.00)	3(0.05)	43(0.70)	12(0.20)	0(0.00)	0(0.00)	0(0.00)	4(0.07)	0(0.00)	0(0.00)	0(0.00)	70(1.15)
L. striatus	39	10(0.26)	0(0.00)	1(0.03)	6(0.15)	13(0.33)	0(0.00)	0(0.00)	0(0.00)	0(0.00)	0(0.00)	0(0.00)	0(0.00)	0(0.00)	30(0.77)
Praomys spp.	18	1(0.06)	3(0.17)	0(0.00)	0(0.00)	0(0.00)	1(0.06)	0(0.00)	0(0.00)	0(0.00)	1(0.06)	0(0.00)	0(0.00)	0(0.00)	6(0.33)
C. gambianus	12	0(0.00)	0(0.00)	0(0.00)	0(0.00)	0(0.00)	0(0.00)	0(0.00)	0(0.00)	6(0.50)	0(0.00)	0(0.00)	0(0.00)	0(0.00)	6(0.50)
Thamnomys spp.	7	6(0.80)	0(0.00)	1(0.14)	0(0.00)	0(0.00)	0(0.00)	0(0.00)	0(0.00)	0(0.00)	0(0.00)	0(0.00)	0(0.00)	0(0.00)	7(1.00)
Unknown	7	3(0.43)	2(0.29)	0(0.00)	2(0.29)	1(0.14)	7(1.00)	0(0.00)	0(0.00)	0(0.00)	0(0.00)	0(0.00)	0(0.00)	0(0.00)	15(2.14)
L. sikapusi	4	0(0.00)	0(0.00)	0(0.00)	1(0.25)	1(0.25)	0(0.00)	0(0.00)	0(0.00)	0(0.00)	0(0.00)	0(0.00)	0(0.00)	0(0.00)	2(0.50)
Mylomys spp.	1	1(1.00)	0(0.00)	0(0.00)	0(0.00)	0(0.00)	0(0.00)	0(0.00)	0(0.00)	0(0.00)	0(0.00)	0(0.00)	0(0.00)	0(0.00)	1(1.00)
Total	3201	1939(0.61)	436(0.14)	413(0.13)	343(0.11)	315(0.10)	249(0.08)	177(0.06)	21(0.01)	38(0.01)	13(0.00)	1(0.00)	1(0.00)	29(0.01)	3975(1.24)

Hosts and fleas are listed in descending order of abundance.

Hosts: *R. rattus: Rattus rattus; A. niloticus: Arvicanthis niloticus; M. minutodies: Mus minutodies; T. valida: Tatera valida; T. emini: Taterillus emini; A. hindei: Aethomys hindei; L. flavopunctatus: Lophuromys flavopunctatus; L. striatus: Lemniscomys striatus; C. gambianus: Cricetomys gambianus; L. sikapusi: Lophuromys sikapusi*.

Fleas: Cf: *Ctenocephalides felis;* Cc: *Ctenophthalums cabirus;* Cb: *Cetonphthalums bacopus;* Dl: *Dinopsyllus lypusus;* Dlo: *Dinopsyllus longifrons:* Eg: *Echinophaga gallinaceum;* La: *Leptosyllus aethiopicus;* St: *Stivalius torvus;* Tp: *Tunga penetrans;*Un: Unknown;Xb: *xenopsylla brasiliensis;* Xc: *Xenopsylla cheopis;* Xn: *Xenopsylla nubica.*

**Table 2:** **Number of fleas and number of fleas per host collected from the most common small mammals**

				**Total no. fleas collected (no. fleas/hosts examined)**															
**Host species**		**No. hosts**		**XC**		**Xb**		**Xn**		**DI**		**Cc**		**St**		**Eg**		**Total**	
		Above	Below	Above	Below	Above	Below	Above	Below	Above	Below	Above	Below	Above	Below	Above	Below	Above	Below
R. rattus	In	250	268	1(0.00)	310(1.16)	119(0.48)	0(0.00)	0(0.00)	2(0.01)	9(0.00)	0(0.00)	9(0.04)	0(0.00)	4(0.02)	0(0.00)	0(0.00)	79(0.29)	145(0.58)	415(1.55)
	Out	240	322	0(0.00)	240(0.75)	119(0.50)	0(0.00)	0(0.00)	5(0.02)	3(0.01)	0(0.00)	2(0.01)	1(0.00)	0(0.00)	0(0.00)	81(0.34)	16(0.05)	210(0.88)	268(0.83)
A. Niloticus	In	214	144	0(0.00)	45(0.31)	50(0.23)	0(0.00)	0(0.00)	0(0.00)	168(0.79)	4(0.03)	158(0.74)	2(0.01)	1(0.00)	5(0.03)	0(0.00)	0(0.00)	387(1.81)	59(0.41)
	Out	101	151	0(0.00)	421(2.79)	68(0.67)	0(0.00)	0(0.00)	7(0.05)	83(0.82)	0(0.00)	63(0.62)	1(0.01)	1(0.01)	0(0.00)	0(0.00)	0(0.00)	223(2.21)	433(2.87)
Crocidura spp.	In	169	111	0(0.00)	44(0.40)	14(0.08)	0(0.00)	0(0.00)	0(0.00)	11(0.07)	1(0.01)	9(0.05)	0(0.00)	144(0.85)	1(0.01)	0(0.00)	0(0.00)	182(1.08)	50(0.45)
	Out	61	86	1(0.02)	311(3.62)	5(0.08)	0(0.00)	0(0.00)	0(0.00)	12(0.20)	1(0.01)	2(0.03)	0(0.00)	32(0.52)	0(0.00)	0(0.00)	0(0.00)	53(0.87)	314(3.65)
mastomys spp.	In	62	55	0(0.00)	6(0.11)	0(0.00)	0(0.00)	0(0.00)	0(0.00)	0(0.00)	0(0.00)	0(0.00)	0(0.00)	5(0.08)	0(0.00)	0(0.00)	0(0.00)	5(0.08)	7(0.13)
	Out	47	29	0(0.00)	0(0.00)	0(0.00)	0(0.00)	0(0.00)	0(0.00)	0(0.00)	0(0.00)	0(0.00)	0(0.00)	7(0.15)	0(0.00)	0(0.00)	0(0.00)	7(0.15)	0(0.00)
M. minutodies	In	14	70	0(0.00)	10(0.14)	1(0.07)	0(0.00)	0(0.00)	52(0.74)	6(0.43)	0(0.00)	0(0.00)	0(0.00)	0(0.00)	0(0.00)	0(0.00)	1(0.01)	7(0.50)	63(0.90)
	Out	14	60	0(0.00)	26(0.43)	0(0.00)	0(0.00)	0(0.00)	163(2.72)	2(0.14)	0(0.00)	0(0.00)	0(0.00)	0(0.00)	0(0.00)	0(0.00)	0(0.00)	2(0.14)	191(3.18)
T. valida	In	14	70	0(0.00)	10(0.14)	1(0.07)	0(0.00)	0(0.00)	52(0.74)	6(0.43)	0(0.00)	0(0.00)	0(0.00)	0(0.00)	0(0.00)	0(0.00)	1(0.01)	70(0.50)	63(0.90)
	Out	14	60	0(0.00)	26(0.43)	0(0.00)	0(0.00)	0(0.00)	163(2.72)	2(0.14)	0(0.00)	0(0.00)	0(0.00)	0(0.00)	0(0.00)	0(0.00)	0(0.00)	2(0.14)	191(3.18)
T. emini	In	8	60	0(0.00)	7(0.12)	0(0.00)	0(0.00)	0(0.00)	63(1.05)	3(0.38)	0(0.00)	0(0.00)	0(0.00)	0(0.00)	0(0.00)	0(0.00)	0(0.00)	4(0.50)	75(1.25)
A. hindei	Out	9	80	0(0.00)	3(0.04)	0(0.00)	0(0.00)	0(0.00)	105(1.31)	0(0.00)	0(0.00)	0(0.00)	0(0.00)	0(0.00)	0(0.00)	0(0.00)	0(0.00)	0(0.00)	108(1.35)
L. flavopunctatus	In	37	0	0(0.00)	0(0.00)	1(0.03)	0(0.00)	0(0.00)	0(0.00)	1(0.03)	0(0.00)	22(0.59)	0(0.00)	4(0.11)	0(0.00)	0(0.00)	0(0.00)	28(0.76)	0(0.00)
	Out	24	0	0(0.00)	0(0.00)	7(0.29)	0(0.00)	0(0.00)	0(0.00)	2(0.08)	0(0.00)	21(0.88)	0(0.00)	8(0.33)	0(0.00)	0(0.00)	0(0.00)	42(0.00)	0(0.00)
L. striatus	In	3	8	0(0.00)	0(0.00)	0(0.00)	0(0.00)	0(0.00)	0(0.00)	1(0.33)	0(0.00)	1(0.33)	0(0.00)	0(0.00)	0(0.00)	0(0.00)	0(0.00)	2(0.67)	0(0.00)
	Out	6	22	0(0.00)	10(0.45)	0(0.00)	0(0.00)	0(0.00)	1(0.00)	5(0.83)	0(0.00)	12(2.00)	0(0.00)	0(0.00)	0(0.00)	0(0.00)	0(0.00)	17(2.83)	11(0.50)
Total	In	837	904	3(0.00)	572(0.63)	208(0.25)	0(0.00)	0(0.00)	125(0.14)	213(0.25)	7(0.01)	201(0.24)	3(0.00)	174(0.21)	7(0.01)	0(0.00)	80(0.09)	808(0.97)	834(0.92)
	Out	559	913	1(0.00)	1393(1.53)	238(0.43)	0(0.00)	0(0.00)	288(0.32)	117(0.21)	6(0.01)	109(0.19)	2(0.00)	68(0.12)	0(0.00)	81(0.14)	16(0.02)	622(1.11)	1710(1.87)

Flea infestation numbers are given for above and below 1300 m and separated by collections during the plague season (August-December) or outside of the plague season (January-July). Only the seven most common fleas and ten most common small mammals are listed, but column and row totals include numbers from the less common fleas and hosts not listed. Hosts and fleas are listed in descending order of abundance.

Total small mammal abundance, species diversity, species richness, and the abundance of *R. rattus, A. niloticus*, and *Mastomys spp*. were not significantly different above and below 1300 m averaged across all trapping sessions. However, *Crocidura spp.* were significantly more abundant above 1300 m (0.84 per 100 trap nights) than below 1300 m (0.50/100 trap nights; Wald χ^2^ = 5.24, p = 0.02). Flea abundance per host did not differ significantly above or below 1300 m (Wald χ^2^ = 0.17, p = 0.68). Flea species richness and diversity were both significantly higher above 1300 m than below 1300 m (Wald χ^2^ = 12.49, p = 0.0004; Wald χ^2^ = 43.32, p < 0.0001). Flea species richness and diversity remained higher above 1300 m than below 1300 m both during and outside of the plague season.

### Comparisons of temporal changes in host abundance above and below 1300 m

**Figure 4:**
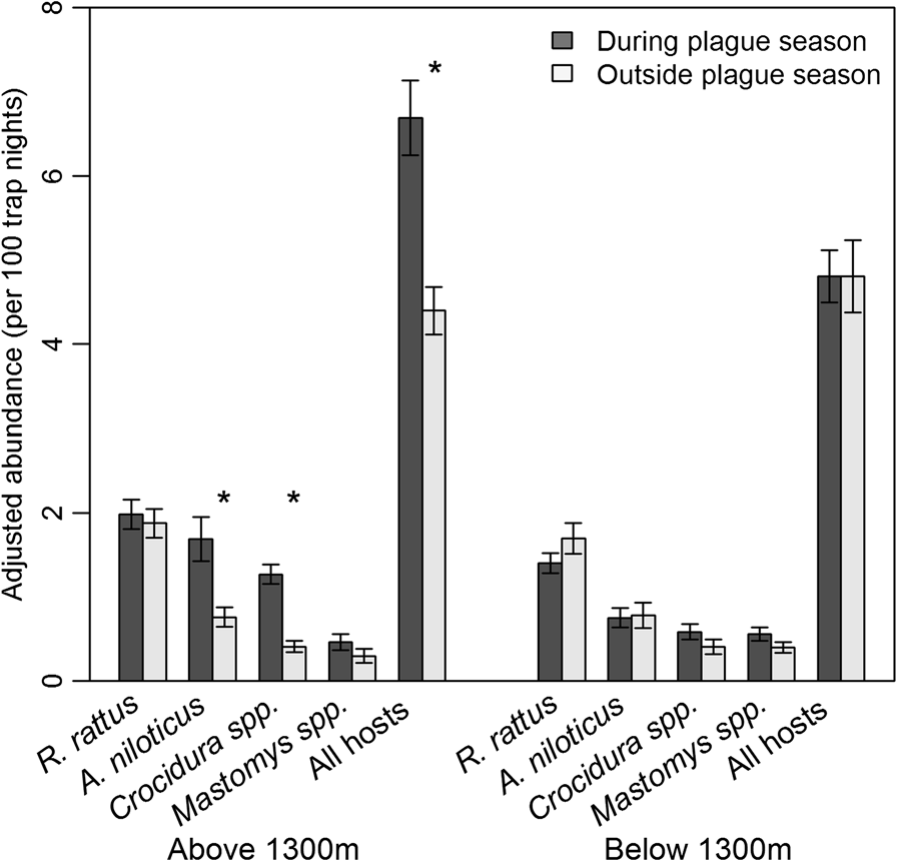
**Comparison of abundance of most common small mammals during and outside of the plague season.** Abundance is mean adjusted abundance per homestead inside and outside of huts (per 100 trap nights). Error bars represent ±1 SE. *Indicates statistically significant difference (α = 0.05) between abundance during and outside of plague season using a generalized linear mixed model with homestead ID as a random effect.

**Figure 5:**
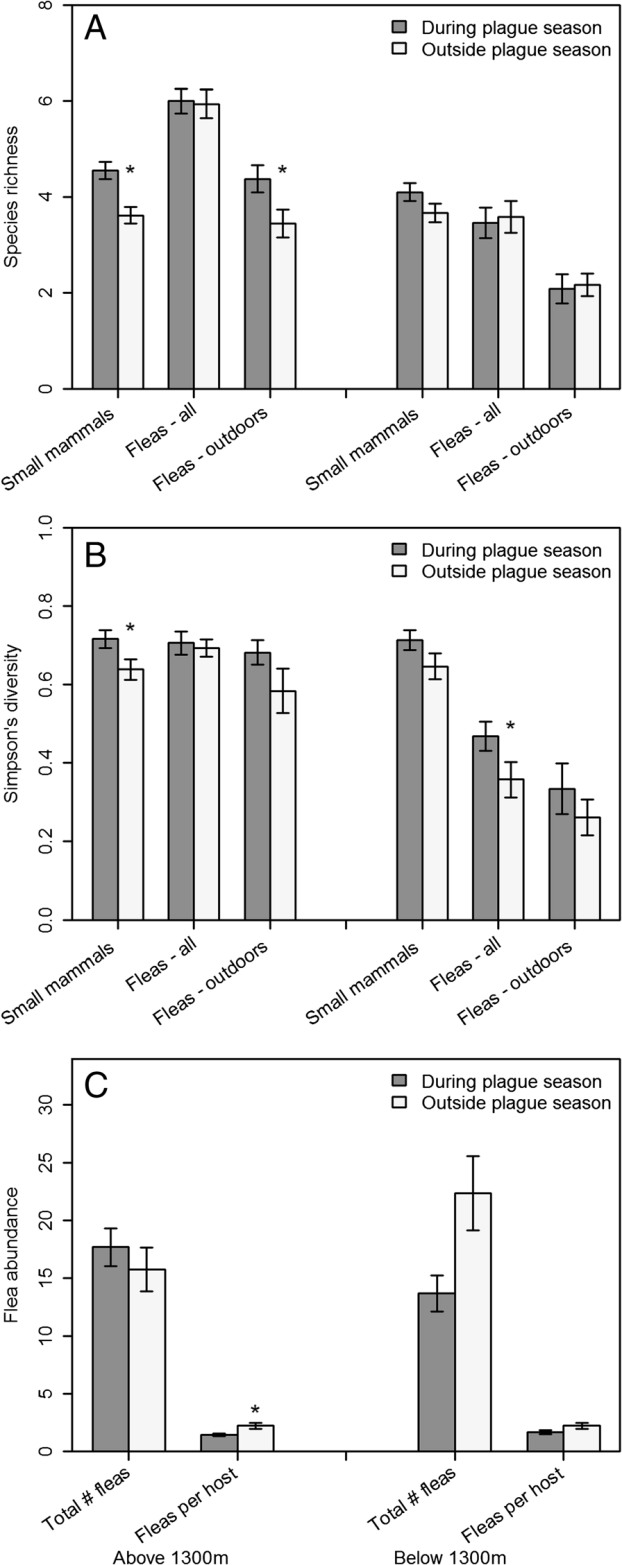
**Species richness, diversity, and abundance of small mammals and fleas inside and outside plague risk area. (A)** Comparison of species richness of small mammals and fleas during and outside of plague season. Fleas-all is the number of flea species collected from both indoors and outdoors in a homestead, Fleas-outside is the number of flea species collected from outside of the huts in a homestead. **(B)** Species diversity of small mammals and fleas during and outside of plague season. **(C)** Total abundance of fleas per homestead and fleas per small mammal host during and outside of plague season. Error bars represent ±1 SE. *Indicates statistically significant difference (α = 0.05) between abundance during and outside of plague season.

Total small mammal abundance was higher during the plague season than outside of the plague season at sites above 1300 m (Wald χ^2^ = 23.55, p < 0.0001), but not at sites below 1300 m (Wald χ^2^ < 0.01, p = 0.99; Figure 4). In addition, the abundance of *A. niloticus* (Wald χ^2^ = 14.76, p = 0.0001) and *Crocidura spp.* (Wald χ^2^ = 43.31, p < 0.0001) were both higher during the plague season than outside of the plague season at sites above 1300 m, but not at sites below 1300 m (Figure 4). These results were significant whether measuring abundance from all trap locations at a site or only from traps placed outside of the huts. There was a significant decrease in small mammal abundance collected from within huts below 1300 m during the plague season (Wald χ^2^ = 6.26, p = 0.01), but no change above 1300 m (Wald χ^2^ = 0.01, p = 0.93). Total *R. rattus* abundance did not differ seasonally at sites above 1300 m (Wald χ^2^ = 0.28, p = 0.60), but *R. rattus* abundance was significantly lower during the plague season below 1300 m (Wald χ^2^ = 4.72, p = 0.03; Figure 4). There was no seasonal change in *R. rattus* abundance within huts, but the number of *R. rattus* trapped outside of the huts varied seasonally both above (Wald χ^2^ = 4.72, p = 0.03) and below 1300 m (Wald χ^2^ = 4.94, p = 0.03). Above 1300 m *R. rattus* abundance outside of the huts increased significantly during the plague season (0.07/100 trap nights outside of season vs. 0.19/100 trap nights during season), but below 1300 m *R. rattus* abundance outside of the huts decreased significantly during the plague season (0.19/100 trap nights outside of season vs. 0.06/100 trap nights during season). However, the number of *R. rattus* collected outside of the huts (n = 82; 0.13/100 trap nights) was relatively small compared to the number of *R. rattus* captured within huts (n = 988; 8.8/100 trap nights). Small mammal species richness and diversity were both significantly higher during the plague season than outside of the plague season at sites above 1300 m (Figure 5; Wald χ^2^ = 17.69, p < 0.0001; Wald χ^2^ = 6.05, p = 0.01), but not at sites below 1300 m (Wald χ^2^ = 3.35, p = 0.07; Wald χ^2^ = 2.61, p = 0.11). There was no significant effect of distance from homestead in the abundance of any of the four main small mammal species either above or below 1300 m. In addition, there was no evidence of any seasonal variations in the effect of distance from homestead on the abundance of any of the four main small mammal species above or below 1300 m.

### Comparisons of temporal changes in flea abundance and diversity above and below 1300 m

Flea abundance per host was significantly higher outside of the plague season than it was during the plague season above 1300 m (Figure 5C; Wald χ^2^ = 7.31, p = 0.007), and showed a non-significant trend towards higher abundance outside of the plague season below 1300 m (Wald χ^2^ = 2.83, p = 0.09). Above 1300 m flea species richness and diversity from all small mammals did not vary seasonally (Wald χ^2^ = 0.03, p = 0.86; Wald χ^2^ = 0.16, p = 0.70), but flea species richness on small mammals collected outside of huts was significantly higher during the plague season than outside of the plague season (Wald χ^2^ = 6.75, p = 0.009; flea diversity: Wald χ^2^ = 2.24, p = 0.13). Below 1300 m flea species richness did not vary seasonally (Wald χ^2^ = 0.16, p = 0.69), but flea diversity was significantly higher during the plague season than outside of the plague season (Wald χ^2^ = 4.18, p = 0.04).

### Associations between small mammal abundance and site-specific climate and crop measures

There was no significant association between the abundance of *R. rattus* above or below 1300 m and any temperature or precipitation variables. There was a positive association between *R. rattus* abundance below 1300 m and the percentage of homesteads harvesting maize or millet five months prior (r^2^ = 0.59).

The strongest single correlations between *A. niloticus* and climate measures above 1300 m were a negative association between *A. niloticus* abundance and the monthly mean temperature at a one month lag (r^2^ = 0.43) and a negative association with monthly precipitation at a six month lag (r^2^ = 0.41). The strongest correlation between *A. niloticus* abundance and a single crop measure was a positive association with the percentage of homesteads harvesting maize or millet in the current month (r^2^ = 0.52). The two best fit models of *A. niloticus* abundance above 1300 m with climate-only variables included a negative association with monthly precipitation at a six month lag and either a positive association with precipitation at a four month lag (r^2^ = 0.50) or a positive association with the current monthly mean temperature (r^2^ = 0.48; Table 3). The best fit model of *A. niloticus* abundance above 1300 m that emerged when crop measures were included in model construction showed a positive association with the percentage of homesteads harvesting maize or millet in the current month and a positive association with the harvest of ground nuts four months prior (r^2^ = 0.69; Table 3).

**Table 3:** **Best fit models of** ***Arvicanthis niloticus*** **abundance above 1300 m with climate and crop variables**

**Model**	**R** ^**2**^	**AIC**	**∆AIC**	**Model parameters**	**Parameter estimate**
*Climate variables only – Above 1300 m*					
1	0.50	−217.3	0	Precipitation – 6 month lag	−0.56
				Precipitation – 4 mo. Lag	0.37
2	0.48	−215.6	1.7	Precipitation – 6 mo. Lag	−1.4
				Mean temperature – 0 mo. lag	0.83
*Climate and crop variable – Above 1300 m*					
1	0.69	−232.3	0	Maize/Millet harvest – 0 mo. lag	0.47
				Nut harvest – 4 mo. Lag	0.53
2	0.68	−231.0	1.3	Maize/Millet harvest – 0 mo. lag	0.51
				Bean harvest – 4 + 5 mo. Lag	0.51

Model r^2^, AIC, and ∆AIC values and parameter estimates for all models with ∆AIC <2 and parameter estimates significant at an α = 0.05 confidence level. ∆AIC represents the difference between a model’s AIC value and the AIC value of the best fit overall model. Parameter estimate values are expressed as a change in abundance per 100 trap nights as a result of a 1°C increase in temperature, 100 mm increase in precipitation, or per each additional homestead at a trapping location harvesting a given crop.

The strongest single correlations between *Crocidura spp.* abundance and climate measures above 1300 m were a positive association with monthly precipitation at a three month lag (r^2^ = 0.44) and a negative association with mean temperature at a one month lag (r^2^ = 0.43). The strongest single correlations between *Crocidura spp.* abundance above 1300 m and crop measures were a negative association with the percentage of homesteads harvesting beans six months prior (r^2^ = 0.34) and a negative association with the percentage of homesteads harvesting millet or maize six months prior (r^2^ = 0.32). The best fit model of *Crocidura spp.* abundance above 1300 m incorporating only climate variables included a positive association with monthly precipitation at a three month lag and a negative association with the current monthly mean temperature (r^2^ = 0.58; Table 4). The best fit models of *Crocidura spp.* abundance above 1300 m incorporating climate and crop variables also included a positive association with monthly precipitation at a three month lag along with either a negative association with the percentage of homesteads harvesting potatoes at a two month lag (r^2^ = 0.60) or a negative association with the percentage of homesteads harvesting pumpkins at a three month lag. (r^2^ = 0.58; Table 4).

**Table 4:** **Best fit models of** ***Crocidura spp.*** **abundance above 1300 m with climate and crop variables**

**Model**	**R** ^**2**^	**AIC**	**∆AIC**	**Model parameters**	**Parameter estimate**
*Climate variables only – Above 1300 m*					
1	0.58	−245.6	0	Precipitation – 3 mo. lag	0.28
				Mean temperature – 0 mo. lag	−0.15
2	0.56	−244.3	1.3	Precipitation – 3 mo. lag	0.41
				Precipitation – 4 mo. lag	−0.25
*Climate and crop variables – Above 1300 m*					
1	0.60	−247.6	0	Precipitation – 3 month lag	0.43
				Potato harvest – 2 mo. lag	−0.32
2	0.58	−245.7	1.9	Precipitation – 3 mo. lag	0.53
				Pumpkin harvest – 3 mo. lag	−0.29

Model r^2^, AIC, and ∆AIC values and parameter estimates for all models with ∆AIC <2 and parameter estimates significant at an α = 0.05 confidence level. ∆AIC represents the difference between a model’s AIC value and the AIC value of the best fit overall model. Parameter estimate values are expressed as a change in abundance per 100 trap nights as a result of a 1°C increase in temperature, 100 mm increase in precipitation, or per each additional homestead at a trapping location harvesting a given crop.

## Discussion

Small mammal abundance, species richness, and diversity increased significantly during the plague season within a region of elevated plague risk in Western Uganda. In particular, the abundance of *A. niloticus* and *Crocidura spp.* increased during the plague season at higher elevations within the region of elevated plague risk, but did not show the same increase in abundance at lower elevations outside of the plague zone. The seasonal abundance of *Crocidura spp*. were positively associated with precipitation at a three month lag, while the abundance of *A. niloticus* was negatively associated with precipitation at a six month lag. In contrast, the abundance of the domestic rat population (*Rattus rattus*) did not show significant seasonal fluctuations along the elevation gradient and there was no association between *R. rattus* abundance and any climate variables.

Small mammal species *A. niloticus* and *Crocidura spp.* have been implicated as significant hosts in enzootic transmission for several reasons. Although the level of susceptibility of these two mammalian taxa to *Y. pestis* infection has yet to be investigated, serological evidence of exposure suggests some resistance to plague-caused mortality exists in their populations [1,18,41] and seropositive *Arvicanthis spp.* and *Crocidura spp.* (as well as *Mastomys spp.* and several other species collected in this study) have been found in other plague foci within East Africa [24,42]. In particular, seropositive *A. niloticus* have been collected from the Ituri region of the Democratic Republic of Congo, which is near our study area and may form a single contiguous plague focus [42]. It also has yet to be determined whether they develop sufficiently high bacteremia to infect feeding fleas but the fact they live in close proximity to human habitations, harbor fleas that are capable of vectoring *Y. pestis*, and harbor fleas (*X. cheopis* or *X. brasiliensis*) that are shared with the presumed epizootic host (*R. rattus*) [25] is noteworthy and suggests they could play an important role in the ecology and epidemiology of plague in the West Nile region. In addition, *A. niloticus* shares similarities to other key enzootic hosts in other plague foci in that they are heavily infested with a diverse flea community, are colonial and burrow-dwelling [43]. Such traits likely facilitate flea transfer amongst hosts and survival of fleas within burrows. Although *Crocidura* spp. generally harbor a lower diversity of fleas, their omnivorous feeding behavior, which includes carrion, could facilitate *Y. pestis* transmission via direct contact [44,45]. Under the assumption that *Y. pestis* transmission rates are density dependent, our observation that the abundance of these two species increased during the plague season within the focus, but not outside of the focus, supports the notion that these hosts are likely important in the transition from enzootic to epizootic transmission. In contrast to the fluctuations in the abundance of *A. niloticus* and *Crocidura spp.*, the abundance of the domestic rat population (*R. rattus*) did not show significant seasonal fluctuations along the elevation gradient, despite the fact that it is an epizootic host and presumably the primary source of infectious flea bites for humans living in this region.

On-host flea species diversity in the West Nile region of Uganda is significantly higher within the plague focus above 1300 m while flea abundance does not appear to vary significantly with elevation [23]. Our results corroborate both of these findings and indicate that the high on-host flea diversity does not vary seasonally and remains significantly higher above 1300 m both during and outside of the plague season. Multiple flea species collected at sites within the plague focus have been reported to be naturally infected with *Y. pestis* and competent vectors under laboratory conditions (e.g., *X. brasiliensis, D. lypusus*)*,* have been found to be naturally infected with *Y. pestis* (*C. cabirus*)*,* or belong to genera containing other species known to be naturally infected or capable of transmitting plague bacteria (*S. torvus)* [1]. The fact that these four species of fleas were mostly absent from small mammals collected outside of the plague focus suggests that a diverse flea community may help maintain the *Y. pestis* transmission cycle within the enzootic community [23]. For example, *X. brasiliensis* was found frequently on both *R. rattus* and *A. niloticus* within the plague focus and may serve as a bridging vector between these two host species. *Dinopsyllus lypusus* and *C. cabirus* were common on *A. niloticus* above 1300 m but not below 1300 m and *S. torvus* was abundant on *Crocidura spp.* above 1300 m but almost entirely absent below 1300 m. Although a study in Tanzania found no difference in flea abundance or diversity between plague-endemic and plague-free areas, hosts that were more abundant in the plague-endemic locations tended to harbor more closely-related fleas, showing the importance of species composition [46]. All three of the previously mentioned flea species were occasionally found on all four of the most common small mammal species, therefore they may maintain transmission within the *A. niloticus* or *Crocidura spp.* populations or also contribute to the maintenance of *Y. pestis* transmission within a network of multiple sylvatic host species.

Unlike flea species diversity, the diversity of small mammals did not vary significantly with elevation, however small mammal species richness and diversity did increase significantly during the plague season within the plague focus. The abundances of both *A. niloticus* and *Crocidura spp.* increased significantly during the plague season within the plague focus leading to a significant increase in the total small mammal abundance within the plague focus. A previous study based on only one year of data found no significant differences in small mammal abundances between sites inside and outside of the plague focus in the region when all trapping sessions were pooled together [23]. These significant seasonal changes in total small mammal abundance and the abundance of several competent *Y. pestis* hosts within the plague focus may be important for promoting an increase in transmission among sylvatic hosts if transmission is density-dependent in this system. An increase in abundance could also increase the number of susceptible individuals in these populations. An increase in the abundance of *A. niloticus* and *Crocidura spp.* could also increase the likelihood of spillover of infectious fleas and *Y. pestis* to the main epizootic host (*R. rattus*) and humans.

Significant increases in the number of *R. rattus* captured outside of huts were observed during the plague season compared with outside of the plague season. In contrast, abundance of *R. rattus* captured inside of huts, which represented the vast majority of captures, remained stable between seasons. These findings suggest that although *R. rattus* living away from homes experience fluctuations in abundance that may track rainfall or crop harvests, those collected in the hut environment do not appear to be limited by environmental conditions or food availability. This would also explain why *R. rattus* abundance was not associated with precipitation or temperature inside or outside of the plague focus. It also doesn’t appear that *R. rattus* abundance is a factor in distinguishing between high and low risk areas within the West Nile region as *R. rattus* abundance didn’t differ between sites inside and outside of the plague focus. It is possible that the abundance of *R. rattus* inside of huts is consistently maintained at sufficient levels to serve as epizootic hosts. Indeed, looking at households within the plague risk area, no significant differences in rat abundance were observed between villages with a history of human plague cases and those without [47]. However, the increase in abundance of *R. rattus* outside of the huts during the plague season within the focus may increase contact rates with other small mammals and promote the spillover of fleas and *Y. pestis* from sylvatic hosts such as *A. niloticus*, *Crocidura spp*. or *Mastomys spp*.

The negative association of *A. niloticus* with precipitation at a six month time lag indicates that *A. niloticus* abundance increases six months after the middle of the dry season and is lowest several months following the end of the rainy season. In addition, *A. niloticus* abundance was also positively associated with either precipitation at a four month lag or the current monthly temperature. Previous research has associated the inter-annual variation in human plague cases in the West Nile region with below average dry season (December-February) precipitation and above average precipitation in June and July prior to the start of the plague season in August [22]. The negative association of *A. niloticus* abundance with precipitation at a six month lag suggests that low precipitation during the dry season may favor increases in the *A. niloticus* population six months later. This translates to an expected increase in *A. niloticus* during June-August, or just preceding the plague season. This could provide ample time for epizootic transmission within sylvatic hosts followed by spillover into *R. rattus* and later into humans. Additionally, a positive association with precipitation in June and July would correspond to an increase in *A. niloticus* abundance in October and November when plague incidence peaks in the human population. This influx in the *Arvicanthis* population, presumably through births, could provide additional susceptible hosts to perpetuate the *Y. pestis* transmission cycle.

The positive association of *Crocidura spp.* abundance with precipitation at a three month lag would also correspond to an increase in *Crocidura spp*. coinciding with the occurrence of plague in humans. A positive association between precipitation and the incidence of plague in humans can result from a trophic cascade where rainfall promotes the growth of food sources for the sylvatic hosts followed by an increase in their reproduction and population size [9,10]. Increases in the population density of host populations have been associated with plague epizootics in rodent populations and an increased risk of spillover to humans in several systems [7,48-50]. *A.niloticus* is capable of giving birth to litters of 4–12 young every 23 days during the breeding season and can breed year-round under highly favorable conditions [43]. The high reproductive rate of *A. niloticus* suggests that the population could increase within six months of the height of the dry season and several months after the onset of the first rainy season as a response to an increase in plant primary productivity.

The abundance of *A. niloticus* was positively correlated with the harvest of millet and maize in the current month. This positive association without a time lag suggests a behavioral, rather than a reproductive, response to the harvesting of millet and maize. *A. niloticus* may be drawn to the millet that is left to dry outside of the homesteads as well as the grains that are dropped in the field following the harvest. The abundance of small mammals in a plague-endemic region of Tanzania also appear to be positively influenced by the harvesting of maize and potato [51]. We did not see a significant shift in relative abundance along the transect lines outside of the huts, which would have suggested a strong shift in the population distribution into the peridomestic area next to the homestead. Because the transect lines only extended 40 meters out from the huts, the increase in abundance along the entire transect suggests that individual *A. niloticus* may be moving from the sylvatic environment into the crop fields surrounding the homesteads within the plague focus. The peak timing of the combined millet and maize harvests is August and September, which coincides with the beginning of the plague season. This provides further evidence that the timing of epizootics in the domestic *R. rattus* population may be at least in part driven by more frequent contact with enzootic hosts such as *A. niloticus. Crocidura spp.* abundance was not strongly associated with the current month’s harvest of any crops, but was negatively associated with the bean, millet, and maize harvests at a six month lag suggesting that the increase in *Crocidura spp.* abundance during the plague season was not related to the maize or millet crop harvest at that time. It is possible that *Crocidura* only responds indirectly to crop harvesting since they eat primarily insects, which may increase their populations following the harvest.

The seasonal increase in the abundance of key potential host species within the area of elevated plague risk, but not outside of this area, suggests that changes in small mammal abundance may create favorable conditions for epizootic transmission in the West Nile region of Uganda. This supports the previous hypothesis that sylvatic small mammal species are essential for maintaining *Y. pestis* within the long standing plague foci in East Africa [18,52]. In particular, the increased abundance of *A. niloticus* and *Crocidura spp.* within the plague focus during the plague season supports previous suggestions that these species are important hosts of the *Y. pestis* transmission cycle in this region and may play a large role in the timing of epizootics. The lagged response of *A. niloticus* and *Crocidura spp.* to precipitation four to six months prior, along with the positive association between *A. niloticus* abundance and the peak harvesting of millet and maize in August, could explain why epizootics and plague transmission in humans typically peak from September through December. In addition, the higher flea diversity at sites above 1300 m may play an important role in maintaining *Y. pestis* transmission cycles within the West Nile plague focus. One limitation to any interpretation of our results is that our collections occurred during a two year period when no large epizootics or epidemics were observed in the West Nile region. Therefore, we cannot describe how small mammal and flea populations would respond during epizootic events. Obviously, it is important to observe these suspected enzootic communities over time to monitor changes prior to and during an epizootic in order to determine which small mammal species and their associated flea communities are critical to the enzootic maintenance and spillover of *Y. pestis* in the West Nile plague focus.

## Conclusion

The increase in abundance of several key small mammal species during the plague season in areas of high plague risk, but not areas of low plague risk, supports previous suggestions that these species are important hosts of the plague transmission cycle in this region and may play a large role in the timing of outbreaks. The abundance of both of these small mammal species was associated with rainfall and the abundance of the most common wild rodent (*Arvicanthis niloticus*) was also positively associated with the harvest of millet and maize. These associations were consistent with previous models of the timing of human plague cases in relation to precipitation in the West Nile region, suggesting that the reproductive or behavioral responses of key small mammal species to these environmental drivers may drive plague risk in Uganda.
